# Dementia in Turkey–Physician's Perspectives on Facilitating and Challenging Aspects in the Diagnostic Process

**DOI:** 10.1002/gps.70068

**Published:** 2025-03-13

**Authors:** Till Neugebauer, Patrick Brzoska, Hilal Özcebe, Yüce Yilmaz‐Aslan

**Affiliations:** ^1^ Faculty of Health Health Services Research School of Medicine Witten/Herdecke University Witten Germany; ^2^ Department of Public Health Faculty of Medicine Hacettepe University Ankara Turkey

**Keywords:** challenges, dementia, dementia diagnosis, facilitating aspects, qualitative

## Abstract

**Objectives:**

Facilitating and challenging aspects of a non‐linear diagnostic process of dementia were explored in interviews with physicians from outpatient clinics of a major hospital in Turkey.

**Methods:**

Semi‐structured interviews were conducted with 15 physicians between March and April 2023. Purposive sampling was used to identify clinics that perform dementia diagnostics, including neurology, geriatrics, and psychiatry outpatient clinics. Interviews were audio‐recorded, transcribed, and analyzed using qualitative content analysis.

**Results:**

Facilitating and challenging aspects were perceived on three levels: (1) diagnostic procedures and methods, which focus on the assessment of dementia and the aspects that arise in the process; (2) communication, which describes the verbal and nonverbal relationships between health care professionals, patients, and their caregivers, as well as their communication styles; (3) care environment, which describes the various environmental influences on patients, both on a sociocultural and institutional level.

**Conclusion:**

The diagnostic process for dementia involves several interrelated aspects, making a flexible, holistic approach essential. In this study, the social and cultural context of the patients and the involvement of the family emerged as crucial elements. By integrating these aspects, along with a mix of informal and formal communication between healthcare providers and families, the diagnostic process can become more patient‐centered and effective. To further improve outcomes, raising awareness and providing education about dementia could reduce stigma and encourage earlier recognition and better management of the condition during the diagnostic process.

**Summary**

- The diagnosis of dementia is viewed as a multi‐faceted process, in which different aspects needed to be considered to provide comprehensive care.
- Sociocultural aspects like religious or traditional beliefs, could lead to stigmatization and further challenges in diagnostics.
- A lack of understanding about dementia and low health literacy among patients and relatives can delay diagnosis, emphasizing the need for improved education and communication strategies.
- A dynamic, iterative, and culturally sensitive approach to dementia diagnostics is recommended.

## 1 Introduction

Dementia is a growing problem in Turkey, with increasing prevalence rates in both urban and rural areas [1, 2, 3]. Due to the lack of a cure, early diagnosis and comprehensive care are necessary to improve quality of life and well‐being of patients by slowing the progression of the condition through early intervention [4, 5]. While the diagnosis of dementia in the international context is typically performed in primary care settings, hospital outpatient clinics play a crucial role in this regard in Turkey. As health care resources are often allocated to these facilities, most people in Turkey consider these clinics as their first point of contact for dementia symptoms [6]. Nevertheless, diagnosing, treating and following up on dementia can be challenging due to the unpredictable nature of the disease [7].

Traditional diagnostic approaches often focus solely on identifying symptoms [8]. This can be problematic if other important aspects, such as the cultural or social context of people with dementia is overlooked. For example, illness perceptions can influence how people with dementia behave during the diagnostic process [9]. A non‐linear diagnostic approach that takes into account patients' expectations, care preferences, and a caregiver assessments to incorporate the experiences of families is therefore recommended to ensure a comprehensive diagnostic process [10]. In Turkey, where family‐centered care is a prevalent practice and its integration can therefore be a significant aspect, it is uncertain to what extent these diverse elements are taken into account when implementing this approach. As a result, challenges may arise during the diagnostic process if patients' needs (e.g., behavior and mental state needs or social interaction needs) are unmet [11]. The difficulty for practitioners is to identify these needs and to include them in the diagnostic process. The limited data available for Turkey show challenges related to lack of knowledge about dementia among healthcare professionals, inadequate training, and limited use of dementia‐specific tests and procedures [12, 13, 14]. In Turkish communities abroad, such as in Germany, contextual factors such as cultural attitudes and non‐acceptance of diagnoses further challenge the diagnostic process [15]. These challenges suggest that the needs of people with dementia are not always sufficiently considered within a comprehensive diagnostic process that goes beyond the mere detection of dementia and also reflects the person's life circumstances by incorporating them into the diagnosis. In order to deal with these challenges, the literature mentions multi‐ and interdisciplinary cooperation, technical resources (e.g., for the analysis of biomarkers), the use of standardized diagnostic procedures, or the introduction of national strategies for dealing with dementia as facilitating aspects [16, 17, 18].

Despite existing research on dementia diagnosis in Europe, there is a notable gap in understanding the specific challenges and facilitators within the Turkish healthcare context. This gap is critical given the increasing prevalence of dementia in Turkey. To date, there has been no examination of Turkish physicians' perceptions and approaches to dementia diagnosis, particularly from a non‐linear perspective that integrates the patient's socio‐cultural context. This study aimed to address this gap by exploring these facilitating and challenging aspects based on a non‐linear understanding in the diagnostic processes of dementia in Turkey and to gain insights in the behaviors of patients, families, and physicians during this process. The physicians' perceptions also provide information about possible reasons for the challenges identified and how they deal with them. Derived from these perceptions we aimed to provide recommendations for improving the diagnostic process of dementia based on the observations and experiences of the interviewees.

## 2 Materials and Methods

This exploratory study used a qualitative approach to address the research objective. Facilitating and challenging aspects of the diagnostic process for people with dementia were identified through semi‐structured interviews with 15 physicians. The interviews reflect the interviewees' subjective experiences and perceptions and thus provide an insight into the diagnostic processes in their practice.

### 2.1 Setting and Participants

The study was conducted in the outpatient clinics of a university hospital in a major Turkish city. Given the central role of the clinical setting in diagnosing dementia in Turkey, purposive sampling was used to ensure that the study targeted the most relevant departments for dementia diagnosis. For this, the university hospital's website was screened for the keyword dementia and departments that explicitly offer dementia clinics were contacted. Verification of their relevance in the context of diagnosing dementia was confirmed by the heads of the departments. As a result, contrasting in this study took place via the selection of medical departments in whose outpatient clinics dementia diagnosis takes place on a regular basis. In the facility surveyed, these were the departments of neurology, geriatrics, and psychiatry. This contrasting approach ensured that the participants were familiar with diagnostic methods, as these make up a large part of their clinical work in these departments. In the geriatric outpatient clinic, around 9–10 patients with dementia were seen per week. Initial exams last 40–60 min, followed by 1‐h cognitive testing with a psychologist and another 1‐h session for family education and care planning. Dementia evaluations occur daily in a dedicated polyclinic room, supported by an interdisciplinary team including a psychologist, social worker, geriatric nurse, occupational therapist, physiotherapist, and nutrition specialist, with psychiatry faculty involvement. The psychiatric outpatient clinic sees dementia patients in the geropsychiatry clinic (8–10 cases weekly), with 1–1.5‐h initial evaluations and 30–60‐min follow‐ups. Patients may be referred to neurology or geriatrics, and psychologists conduct cognitive tests. The neurology outpatient clinic operates a dementia clinic once a week, handling 25–30 dementia cases. Initial evaluations last 45–50 min, with 25–30 min for follow‐ups. A multidisciplinary approach integrates neurology specialists in neurodegenerative diseases, clinical psychologists, and cognitive rehabilitation specialists. Dementia patients are jointly managed with psychiatry and geriatrics, highlighting the critical role of these departments in dementia diagnosis.

Physicians were included into the present study if they were currently working at one of the three outpatient clinics and had the competence to independently diagnose dementia, including doctors in residency training at the time of the interviews as well as fully trained specialists. In addition, English language proficiency to a degree that allowed interviews was necessary. The recruitment of participants was facilitated by the department heads in order to reach as many physicians as possible who meet the inclusion criteria. The final study population consisted of 15 physicians (Table 1) of whom 8 were in residency training and 7 were specialists. Two of the seven specialists were undergoing further training to become geriatric specialists. All participants confirmed their experience with diagnosing dementia. The heterogeneity of experience levels in the sample was not seen as a disadvantage concerning the explorative research objective of this study. Rather, it was viewed as an opportunity to include unbiased approaches and perspective of younger physicians in exploring facilitating and challenging aspects. Due to the institutions function as a university hospital, both residents and specialists were also involved in research processes.

**TABLE 1:** Characteristics of the participants.

Participant	Sex	Age	Clinic	Specialization	Total experience (years)	Experience at clinic
P1	Female	25	Geriatric clinic	Internal medicine resident	1	1 week
P2	Female	32	Geriatric clinic	Geriatric medicine residency	5	5 months
P3	Female	35	Geriatric clinic	Geriatric specialist	11	2 years
P4	Male	29	Psychiatric clinic	Psychiatric resident	5	4 years
P5	Male	34	Geriatric clinic	Internal medicine specialist	5	8 months
P6	Female	38	Geriatric clinic	Geriatric specialist	13	3 years
P7	Female	34	Geriatric clinic	Geriatric medicine residency	11	3.5 years
P8	Male	32	Geriatric clinic	Internal medicine specialist	9	5 months
P9	Female	33	Geriatric clinic	Internal medicine specialist	9	3 years
P10	Female	28	Neurology clinic	Neurology resident	3	3 years
P11	Female	27	Neurology clinic	Neurology resident	3	2 years
P12	Female	26	Neurology clinic	Neurology resident	1	10 months
P13	Female	27	Neurology clinic	Neurology resident	1	3 months
P14	Male	27	Neurology clinic	Neurology resident	4	2.5 years
P15	Female	28	Neurology clinic	Neurology resident	4	3 years

### 2.2 Data Collection

Interviews were conducted between March and April 2023 and lasted 20–61 min. Two two‐person interviews (P1 with P2, P12 with P13) and 11 one‐person interviews were held. Although participant P1 had only started her residency training shortly before the time of the interview, she was interviewed together with a more experienced doctor to take her perceptions into account as well. Data saturation was achieved with 13 interviews. All interviews were conducted by one researcher (T.N.) in either face‐to‐face (in meeting/lecture rooms of the outpatient clinics) or online (via Zoom) format. There was no prior relationship between the interviewer and the interviewees. As this study was part of the first author's master's thesis, which was completed during a research stay in Turkey, and therefore limited resources (e.g., Turkish interviewers) were available, the interviews were conducted in English. Recognizing that English was not the native language of the participants, they were given the opportunity to use online dictionaries to help them express their perspectives.

Before the interviews, the interviewees were given an information letter explaining the reasons for the study and its objectives. During interviews a semi‐structured interview guide was used, developed by the main researcher and reviewed by the research team in a reflexive process. We used a narrative question format to encourage respondents to speak openly about their experiences and to describe situations in which they perceived challenges and how they handled them. The final interview guide consisted of three thematic parts for the main questions: (a) assessment and diagnosis; (b) challenges and facilitating aspects; (c) communication. The interview guide can be found in the supplementary information.

All interviews and transcriptions were carried out by the first author, who, in his academic position as a health services researcher, has gained extensive experience in conducting and analyzing qualitative interviews. Pseudonymization was used as part of the transcription process. Field notes were taken to capture additional content of particular interest about the setting or process of the interviews, such as when interviewees were in a hurry or gestures made during explanations.

The study was approved by Hacettepe University Health Sciences Research Ethics Committee (GO 23/131). The participants were informed about the purpose of the study, the procedure, and their right to withdraw their consent to participate in the study. They confirmed their participation by giving written informed consent prior to the interviews.

### 2.3 Data Analysis

The data material was analyzed by means of qualitative content analysis [19]. For this purpose, a category‐based analysis of the data material was performed in several steps using the software Casquada (version 0.9.1).

The main categories were first developed through a deductive approach, and then refined through a review of 4 selected interviews. Inductive categories were added to capture themes that emerged from the data. The final category system included three main categories: diagnostic procedures and methods, communication, and care environment. Subcategories were developed through open coding with a focus on identifying facilitating and challenging aspects.

Category‐based data analysis involved evaluating relationships between main and subcategories across cases to capture the different narratives of the participants. Due to the explorative design, the entire data material was treated as equivalent and not weighted according to the experience of the interviewees to allow for a comprehensive identification of facilitating and challenging aspects. The coding of the data material was carried out by the first author and was consolidated by means of an iterative process in close cooperation with the other co‐authors. In a final step the findings were integrated into the diagnostic process and strengths in dementia diagnosis were synthesized from the identified facilitators and challenges. These were used to discuss the findings and derive recommendations for improving the diagnostic process of dementia, with a focus on the healthcare context in Turkey.

## 3 Results

Analysis of the data identified both facilitating and challenging aspects of the dementia diagnostic process in the following areas: (1) *diagnostic procedures and methods* focusing on the assessment of dementia and related aspects; (2) *communication* describing verbal and non‐verbal interactions between health professionals, patients, and caregivers; (3) *care environment* considering socio‐cultural and institutional influences that affect the diagnostic process. Table 2 provides a comprehensive overview of these facilitating and challenging aspects.

**TABLE 2:** Facilitating and challenging aspects in dementia diagnostics.

	Facilitating aspects	Challenging aspects
Diagnostic procedures & methods	Resistance resources based on experiences Standardized tests & methods Holistic diagnostics Collaboration with health experts In‐house hospital rotation program Intuition Interest in learning about dementia Education of patients and relatives	Fear of dementia Generalization of symptoms Application of tests Lack of medical exam time Limited follow‐up appointments No need for interprofessional collaboration Influence of comorbidities Differentiation of dementia subtypes Lack of treatment options Denial of symptoms Aggression against health personnel Adherence to treatments Dementia illiteracy
Communication	Informal communication Using familial addressing Building trust, comfort, and a personal connection Physical contact	Crossing professional boarders Unfocused communication
Care environment	Information and awareness campaigns Empathy and understanding Professional distance Family care tradition Recognition of the importance of family involvement Informative meetings	Limited specialist resources High diagnostic costs Geographical barriers Inequality in access Clash of expectations Stigmatization Gender and caregiver roles Burden on family caregivers Lack of caregiver skills Changing family dynamics

### 3.1 Diagnostic Procedures and Methods

The study reveals that physicians' understanding of dementia is primarily shaped by clinical training and personal experiences. The prevailing view is that dementia is a neurodegenerative disease, which is often generalized as Alzheimer's disease.

[…] when I think about dementia, I always recode all information and the only information that comes into my mind is Alzheimer. Alzheimer is a type of dementia and I think, I’m generalizing the dementias like Alzheimer.

(P4)

In contrast, physicians with personal experiences, such as having family members with dementia, have a broader, more empathetic perspective, emphasizing the social and emotional needs of patients.

The first thing that comes into my mind is my grandmother probably. Because she suffered from dementia for many years and I grew up seeing this situation, my parents dealing with this. […] it was really emotionally hard for me.

(P14)

Standardized tests and methods facilitate dementia diagnosis, though challenges remain, particularly concerning the facilities' capabilities and patient responses. Despite standardization, the diagnostic process is complex due to comorbidities and differentiating subtypes. Physicians often rely on clinical intuition and experience for accurate diagnoses.

Most of the time we are smelling it […] it is not important to have names for the disorder. We are trying to find what kind of smell we are having from that patient.

(P15)

A comprehensive diagnostic approach, which includes various aspects of patient history and symptoms, is perceived as important in this context.

When our first examination does not result for it (dementia) […] we ask about everything a geriatric patient could have. Like nutrition and sleep problems or daily activities, incontinence, falls, osteoporosis. We ask every part of it. So, that time they actually tell us they have a problem with it.

(P2)

This method includes various aspects in patient assessment, such as family history and minor indications of dementia symptoms from patients' narratives.

However, time constraints, especially in the Turkish healthcare system, are perceived as challenging. Neurological clinics often limit diagnostic time to 5 min, while geriatric clinics may allow up to an hour, which is still considered insufficient.

The patients need more interview time, I think. […] If you think of the whole country, the interview time is just five or ten minutes. And you just say ‘You have a dementia, you have to take these pills.‘, it’s over. But you don’t understand how patients understand that or what patients feel.

(P4)

We give them thirty minutes, but it’s barely enough, thirty minutes to one hour. But it is not enough because they have so many comorbidities and so many drugs. So, explaining them about all of their diseases, all of their drugs and their exercises, their nutrition. It doesn’t work, it is not enough.

(P2)

Physicians in our study therefore face a conflict between providing comprehensive diagnostics and managing the high patient volume. This conflict in addition to time constraints often results in diagnostics being limited to a single visit, potentially missing important information.

Collaboration and cooperation were seen as beneficial for dealing with this situation. For example, professors play a crucial role in complex cases, offering extensive knowledge and support, particularly to young physicians:

They’re working with you and they assess the patient with us. They see the patients and they help us do the plan, […] which test is needed for the patient.

(P12)

Including different perceptions and experiences by other professionals can help to gather important information, despite the challenges in diagnostics.

### 3.2 Communication

The interviewed physicians emphasize the critical role of relatives in the dementia diagnostic process due to the neurodegenerative nature of the disease. Since patients often experience memory loss and cognitive impairments, they may struggle to communicate their experiences effectively, making it challenging to confirm a diagnosis without input from family members.

One physician noted: Sometimes we have no chance to talk with the patient […] If the disease is very severe, we can’t talk with the patient, and we only sometimes make eye contact only. (P3). This highlights the difficulty when patients attend appointments alone, as communication is vital for accurate diagnostics. In such cases, physicians must rely on written notes or instructions to involve family members:

I told him about the disease, […] and I wrote a paper like this, for example, ‘your son or daughter must come here because we must give some extra information to you and your disease’ and I gave him, but I also knew that he will forget all the spoken and all the things we talked about.

(P5)

Physicians often prefer to speak directly with family members to convey necessary information, acknowledging that patients may lack confidence in their communication abilities. One physician noted that patients “think that their communication signals are not enough” (P7), underscoring the need for family involvement to bridge communication gaps.

Communications with people with dementia involves verbal and non‐verbal interactions, as well as formal and informal styles. Cultural nuances, such as using familial terms like “uncle” or “aunt” are employed by Turkish physicians to create a more comfortable atmosphere, easing patient anxiety and encouraging cooperation. Empathy and compassion are crucial for building rapport, as one participant explained:

Sometimes I said it, we have some dementia people in our family. […] We had this too and we know it’s so difficult. […] You are not alone.

(P15)

However, some physicians caution that overly informal communication may blur professional boundaries and detract from essential diagnostic details. Balancing empathy with the need for clear, direct communication is essential. Physicians often communicate diagnoses “straightforwardly” (P14), blending formal and informal styles to ensure effective diagnostic processes. As one physician stated: “I tell the patient ‘I am asking you a question, only answer the question. We don't need the other sentences.’” (P3), illustrating the need to maintain focus during diagnostics.

### 3.3 Care Environment

The care environment plays a crucial role in the dementia diagnostic process, with several aspects impacting diagnosis. Accessibility to care is perceived as a significant challenge due to the limited availability of specialized services and high diagnostic costs. Diagnoses often occur in large hospital outpatient clinics or private facilities, limiting widespread accessibility in Turkey.

You have to perform a lot of tests and imaging things, but in Turkey it's a little bit expensive, you know. And you have to find the right center for this diagnostic testing.

(P4)

Inadequate public knowledge about dementia exacerbates these accessibility issues. Participants stressed the need for better patient and family education to manage expectations and improve decision‐making. The lack of awareness often leads to misunderstandings and complicates the therapeutic relationship between healthcare providers, patients, and their families. Empathy and support are crucial, especially given the limited treatment options, which can cause feelings of helplessness among patients and caregivers:

I don’t have a holy skin to change the dementia progress, I’m just a doctor and you know, there are a lot of disorders. We are so sorry not to have treatments for that.

(P15)

Sociocultural values were mentioned as influencing the diagnostic process. Traditional beliefs, such as attributing dementia to divine will or considering it a natural part of aging, contribute to stigma and underutilization of diagnostic services.

In Turkey patients believe in god and the diseases are coming from god, […] Yes, yes. God made. Also he kills. God wants that they are dying.

(P3)

Gender roles also impact dementia diagnosis, as caregiving expectations and decision‐making often fall disproportionately on women, influencing timely access to medical care when responsibilities in families are unclear.

P13: […] In our population, generally, the men are more important in total. The daughter is, the daughter and the mother, the wife gives herself to the husband or the son or the father. But not the other way around. P12: When the parents are ill, everyone thinks that daughters must be; P13: The caregivers, must be the caregiver. P12: She also thinks it is her work and she must act like this.

(P12,P13)

Family caregiving, while crucial for facilitating healthcare access, presents emotional and practical burdens. Families often lack the training to manage dementia care effectively, leading to delayed diagnosis as they might try to conceal uncertainties:

Just as I said it could be hard for the relatives because sometimes the dementia patients need caregivers and sometimes the providing of caregiving could be hard for relatives if they are working people. They have to find someone to take care of their parents.

(P10)

The increasing use of external care services or nursing homes reflects changing family dynamics and the growing demands on caregivers, influencing diagnostic decisions and patient outcomes:

We tell them that this patient cannot stay alone that you have to find someone to take care of them or you have to take them with you in your home. And some families are so supportive that they dedicate their life. We have like wifes and husbands that dedicate their life for years to the patients.

(P2)

The physicians view family involvement as a cornerstone of dementia diagnostics, bridging communication gaps and supporting patients throughout their healthcare journey. Families act as advocates, facilitating access to medical care and providing essential information to healthcare providers, with their firsthand knowledge proving invaluable in understanding symptoms and applying diagnostic measures.

### 3.4 Strengths in the Diagnostic Process of Dementia

Based on the facilitating and challenging aspects we condensed eight strengths from the data material: increased awareness and education, comprehensive and holistic care, enhanced collaboration, targeted research and treatment, patient and caregiver support, supportive but focused communication, cultural sensitivity, family‐centered care. These strengths highlight the need for a multilevel and non‐linear approach to dementia diagnosis. Physicians in our study perceived that framework conditions are an important part of the diagnostic process, taking into account the diverse needs of people with dementia.

## 4 Discussion

To our knowledge, this is the first study that uses a qualitative approach to explore physicians' perspectives and subjective views on dementia diagnosis in Turkey. The identified facilitating and challenging aspects identified underscore the critical need for increased public awareness and education about dementia to facilitate early diagnosis. The study found that despite advances in standardized testing and research, time constraints in clinical settings remain a barrier to accurate and timely diagnosis. Collaboration, communication and consideration of the cultural and social context of people with dementia emerge as critical to improve diagnostic accuracy and care coordination.

The findings suggest that many patients and their families have limited knowledge about dementia. A general lack of understanding about dementia and health care in Turkey [20], especially among family members, can create challenges that hinder the diagnostic process. This can contribute to the stigmatization of dementia, resulting in delayed diagnosis. Denial of symptoms and acceptance of the diagnosis were also identified as challenges in this context. This behavior is consistent with other studies that have found challenges in the diagnostic process among persons with a Turkish migratory background in European countries [15, 21, 22, 23]. Consequently, healthcare professionals need to address this lack of acceptance after disclosure of the diagnosis by educating patients and their families about dementia, which may lead to additional burdens. Pre‐diagnosis education could help patients and their families recognize symptoms and seek care earlier. Notably, while there appears to be an increasing interest in information about dementia in Turkey [24], national strategies to address knowledge gaps among the population are still lacking [25]. In our study, participants had a good understanding of dementia, likely due to the specialized expertise available in their outpatient clinics. As physicians are in direct contact with people with dementia and their families, they can provide information to compensate for deficits in knowledge about dementia. In this context, general practitioners and family physicians in ambulatory practices should also be sensitized for dementia and trained in early symptom recognition to ensure comprehensive diagnosis and care for a broader population [12].

Interviewees emphasized the importance of standardized testing for accurate diagnosis, in line with established dementia care guidelines. However, the study found that time constraints affected the thoroughness of the diagnostic process, echoing findings from other studies in which time constraints reduced diagnostic accuracy [26, 27, 28]. Time constraints can lead to diagnostic uncertainty in decision making [27], making collaboration with different professions necessary for our participants to overcome this challenge. The benefits of multi‐ and interprofessional collaboration have been extensively discussed [29, 30], but also in dementia diagnosis and care, collaboration can facilitate a multiprofessional and interdisciplinary care environment [16, 31, 32]. For this, it seems necessary to clarify responsibilities in the diagnostic process. While in our study collaboration was seen as an important part of the diagnosis, other studies on this topic show that responsibilities are not always clear, especially in the clinical setting [14, 33]. In this regard, the involvement of patients and families can be seen as beneficial [34].

Effective communication is essential to successful dementia diagnosis, and different methods are used depending on the situation. Informal communication, such as using familiar terms like “mother” or “uncle”, is emphasized to facilitate patient and family support. This approach is specific to the Turkish language and culture [35, 36]. Physicians reported that this style improved patient participation in diagnosis. A Dutch study by Schinkel et al. [37] found that Turkish migrants preferred communication that addressed social and emotional needs, using informal terms. However, Gültekin [38] cautions that overly informal communication can compromise professional boundaries, echoing the views of our participants. Our findings highlight the importance of direct communication, especially when disclosing dementia diagnoses, to ensure accuracy. A mix of formal and informal communication seems necessary, as this combination can enhance interdisciplinary communication [39].

Our final key finding is the importance of including sociocultural aspects, values, and beliefs in the dementia diagnostic process. In Turkey, strong family support systems often lead families to provide care for relatives with dementia, influenced by cultural and religious beliefs. The Interviewees mentioned that dementia is often seen as an act of God, which shapes how the disease is understood and managed. This perception is reflected in other studies [40, 41] and often results in a reliance on informal care, reducing the use of formal healthcare services. These cultural beliefs can affect diagnostic and treatment decisions in outpatient clinics as it is shown in our results. Therefore, taking these considerations into account is essential for providing effective, patient‐centered care [18]. In addition, family involvement can be a key strength in dementia diagnostics. Given the cognitive limitations associated with dementia, our clinicians believe that focusing on the patient's social and family environment is beneficial. Research shows that family‐centered care can significantly improve the quality of life for people with dementia, promoting patient autonomy and better health outcomes [42, 43, 44, 45]. To fully realize the benefits of family involvement, support services should be tailored to the specific needs of patients and their families [46]. By tailoring these services, health care providers can ensure that families unable or unwilling to provide care can access necessary resources, while patients without family support receive adequate care.

Our results show that the diagnostic process of dementia is embedded in a comprehensive care process. As described before, a linear and one‐sided view of diagnostics cannot meet the demands of the various needs of people with dementia and their families, but also of the physicians [10]. For this reason, dementia diagnostics should rather be seen within a framework of influences that emphasize the strengths of existing resources and prerequisites. This would allow patients and their relatives to be more involved in the early stages of diagnosis, which could reduce uncertainty and stigmatization. This would also give physicians the opportunity to raise awareness of diagnostics and thus achieve a better outcome. Based on our identified strengths we formulated recommendations for the diagnostic process of dementia (Table 3).

**TABLE 3:** Recommendations for improving the diagnostic process of dementia.

Strengths	Recommendations
Public awareness and education	Implement programs to raise awareness about dementia and reduce fear and stigma. Focus on educating the public about the signs, symptoms, and realities of dementia.
Comprehensive and holistic care	Develop comprehensive care plans that address the full range of dementia symptoms and comorbidities. Emphasize individualized assessments and personalized care.
Collaboration among stakeholders	Foster better coordination and communication among healthcare providers, patients, and family members. Encourage a team‐based approach to dementia care and diagnostics.
Research and treatment development	Support ongoing research into dementia to improve understanding and develop more effective treatments. Focus on creating diagnostic tools for differentiating dementia subtypes.
Patient and caregiver support systems	Establish support networks for patients and caregivers to address denial of symptoms and ensure consistent follow‐up care. Provide resources for caregivers.
Communication clarity and effectiveness	Train healthcare professionals in effective communication strategies. Ensure all communication with patients and families is supportive, straightforward, and patient‐centered.
Cultural sensitivity in care	Tailor dementia care and support services to reflect the cultural and social contexts of patients and families.
Family‐centered care approach	Recognize and support the role of families in care and decision‐making for people with dementia. Encourage family involvement in care planning and provide necessary resources.

### 4.1 Strengths and Limitations

In this study, we conducted interviews with physicians in residency and specialists who were also research assistants at a major hospital in Turkey. This hospital is considered one of the most important university hospitals in the country. Interviewees therefore have good access to a large number of people with dementia and to different steps of the diagnostic process. Despite these strengths, there are some limitations to this study.

The results obtained are the subjective views of the doctors, who are influenced not only by the technical and financial possibilities, but also by the structures of the institution in which they work. For this reason, the results can only provide information about the diagnosis of dementia in metropolitan areas of Turkey where sufficient health care is available. Nevertheless, it can be assumed that some of the challenges identified in this study also play a role in rural regions of Turkey, given the increasing prevalence rates in these regions, or are even more pronounced in some cases (e.g., cultural and religious beliefs). Therefore, our proposed recommendations may have some relevance here as well.

In addition, language skills were a significant limitation, as the respondents were not native English speakers. Although care was taken to ensure that the interviews were conducted with a full understanding of the social context by the researcher, potential misunderstandings may have occurred. Understanding the nuanced meanings of relevant content in the context of participants' experiences was a priority during preparation. This approach is consistent with the principles of cross‐cultural interviewing outlined by Patton [47]. To deal with this limitation, participants were asked during the interviews to express themselves in Turkish if they could not express their thoughts in English. Statements in Turkish were then to be translated into English by a Turkish researcher. However, apart from individual words that were mentioned in the context of situation descriptions in order to understand them, no conversation took place in Turkish. The fact that the interviews were conducted by a foreign researcher can also be an advantage, as it allows an impartial view of the situations and perspectives described.

Furthermore, while the heterogeneity in the experience levels of participants allowed for a broader understanding of different aspects of the diagnostic process, it also introduces a potential limitation. Less experienced physicians, particularly those at the beginning of their residency training, may have had limited exposure to the diagnostic process and may primarily rely on theoretical knowledge rather than practical experience. This could have led to perceptions that are shaped more by idealized guidelines rather than real‐world diagnostic challenges. Conversely, more experienced physicians may have developed heuristics that influence their diagnostic decisions. This variability in expertise could affect the way dementia diagnosis is perceived and reported in our study.

While the study focused on physicians, it underscores the importance of considering the roles of patients, family members, and caregivers in dementia diagnosis. Future research should incorporate these contextual aspects to improve the diagnostic process of dementia.

## 5 Conclusion

In conclusion, the study highlights that the diagnosis of dementia does not follow a strictly linear sequence, but requires a multi‐faceted approach. This non‐linear approach underscores the importance of considering the patient's circumstances and broader framework conditions. Insights from a prominent medical institution in Turkey emphasize that effective diagnosis extends beyond the individual patient to include his or her familiy, health professionals and caregivers. The success of diagnostic efforts depends on the environment and circumstances in which they are conducted, yet current implementation often falls short of comprehensive integration. This study provides recommendations that can support the further development of the dementia diagnostic process in Turkey. Given the importance of improving the quality of life of people with dementia, there is an urgent need for strategies and opportunities to address this vulnerable group. In the context of an increasingly aging population, this study provides a starting point for improving dementia care.

## Ethics Statement

The study was approved by Hacettepe University Health Sciences Research Ethics Committee (GO 23/131).

## Consent

Participants gave written consent to participate in this study.

## Conflicts of Interest

The authors declare no conflicts of interest.

## Permission to Reproduce Material From Other Sources

Not applicable.

## Supporting information

Supporting Information S1

## Data Availability

The data that support the findings of this study are available on reasonable request from the corresponding author.
