# Supplementary material for: Dementia in Turkey–Physician's Perspectives on Facilitating and Challenging Aspects in the Diagnostic Process
Source: Int J Geriatr Psychiatry. 2025 Mar 13;40(3):e70068. doi: 10.1002/gps.70068 (PMC11906901; doi:10.1002/gps.70068)
Supplement: Supplementary file 1 — Supporting Information S1 [file GPS-40-e70068-s001.docx]

**Interview guideline for the study “Dementia Diagnosis in Turkey - Physicians' Perspectives on Hindering and Promoting Aspects in the Diagnostic Process”.**

| **Assessment of dementia** | |
| --- | --- |
| **Orientation phase** | |
| „warm up“ with the participant | - Introducing - Aim of the study: Identifying challenging and facilitating aspects in the assessment and diagnosis process of people with dementia - Note on data privacy and anonymity - Ask for informed consent |
| **Introduction phase** | |
| Transition to content level | Can you tell me how long you work here as a physician? So how old are you then? And how long do you work in this outpatient clinic?  So to my first question:  When you think about people with dementia, what is the first thing that comes into your mind? |
| **Main Questions** | |
| ***Assessment and diagnosis*** | |
| Care Procedere | - Can you explain to me how the diagnosis of dementia is done in your facility? - Can you describe a situation in which the assessment of dementia was particularly difficult for you? |
| Patient Involvement | - How well do you think patients are prepared for a diagnosis?   - How would you describe the knowledge of the patients in regard to Dementia? - What behavior of the patients do you perceive here? How do they react to the diagnosis? |
| Other Aspects | - Which influence do comorbidities have on the assessment of dementia? |
| ***Challenges and facilitating aspects*** | |
| Challenges in assessment | - At what stage of dementia do patients usually come to your facility?   - What do you think? Why Are they coming at this point? - What challenges do you see when dealing with people with dementia? |
| Facilitating aspects | - What supportive aspects do they perceive in connection with the assessment? - (What do you think is good in the diagnosis process?)   -> support of family members etc. |
| ***Communication*** | |
|  | - How does your communication with the patients and their relatives work? - When you remember different situations: Who is talking more during the assessment and why? - What feelings do you perceive in patients/relatives when they tell you about the problems? - How do patients/relatives describe everyday situations? (e.g. emotionally, angry, sad etc.) |
| **End phase** | |
| ***Further questions*** | |
| - Feedback | - Are there any uncertainties you have after this interview? - Is there something you would like to say but couldn’t mentioned it during the interview? |
